# A forum on batteries: from lithium-ion to the next generation

**DOI:** 10.1093/nsr/nwaa068

**Published:** 2020-04-17

**Authors:** Weijie Zhao

**Affiliations:** NSR news editor based in Beijing

## Abstract

The 2019 Nobel Prize in Chemistry was awarded to three pioneers of lithium-ion batteries (LIBs)—Prof. John B. Goodenough at the University of Texas, Prof. M. Stanley Whittingham at the State University of New York and Mr. Akira Yoshino at the Asahi Corporation of Japan, which is a great encouragement to the whole field.

LIBs have been developed for several decades with the progress slowing down and their performances approaching some theoretical limits. On the other hand, new types of batteries or power systems, including solid-state batteries, sodium-ion batteries, lithium-sulfur batteries and fuel cells, are being steadily developed, offering new choices for divergent applications. In this panel discussion chaired by *NSR* editorial board member Huiming Cheng, battery experts gather to discuss the challenges and trends of LIBs, the developments and applications of next-generation batteries, as well as the status quo of the battery research and industry in China.

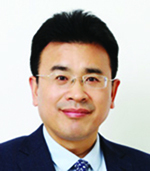

Jun Chen

Professor of the College of Chemistry, Nankai University, Tianjin, China

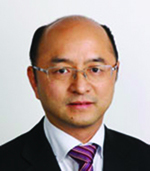

Yunhui Huang

Professor of the School of Materials Science and Engineering, Huazhong University of Science and Technology, Wuhan, China

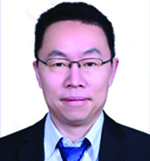

Hong Li

Professor of the Institute of Physics, Chinese Academy of Sciences, Beijing, China

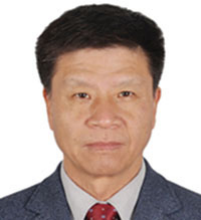

Shigang Sun

Professor of the College of Chemistry and Chemical Engineering, Xiamen University, Xiamen, China

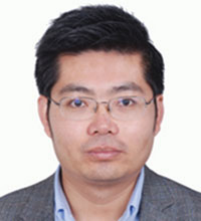

Hongli Zhang

Director of the R&D Institute of Battery, Gotion High-Tech Power Energy Co., Ltd., Hefei, China

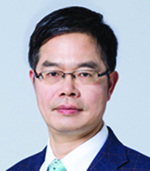

Huiming Cheng (Chair)

Professor of the Shenzhen Geim Graphene Center, Tsinghua-Berkeley Shenzhen Institute, Tsinghua University, Shenzhen, China; Shenyang National Laboratory for Materials Science, Institute of Metal Research, Chinese Academy of Sciences, Shenyang, China

## LIBS ARE REACHING THEIR LIMITS? NOT YET!

**Cheng:** There is an argument that LIBs are approaching their limits. What's your opinion?

**Li:** I personally do not agree. We should evaluate the performance of LIBs from multiple facets, including gravimetric energy density, volumetric energy density, cyclability, charge–discharge rate, temperature adaptability and safety. Among all these facets, only gravimetric and volumetric energy densities have quantitative theoretical limits.

Even if we only consider these two indices, I believe that another 10 years’ research is needed to reach the limits. There are four major types of LIB cathode materials: LiCoO_2_, Li(NiCoMn)O_2_, LiFePO_4_ and LiMn_2_O_4_. Among them, LiFePO_4_ and LiMn_2_O_4_ are approaching their limits, while LiCoO_2_ and Li(NiCoMn)O_2_ still have developing space. The theoretical limits for LiCoO_2_ and Li(NiCoMn)O_2_ are both 274 mAh/g and the current records are ∼205 and ∼210 mAh/g, respectively. By developing high-Ni, low-Co or Co-free Li(NiCoMn)O_2_ and making other improvements, we can further improve their performance and lower their costs.

Besides these four major types, there are also Li-rich cathode materials such as *x*LiMO_2_-(1–*x*)Li_2_MnO_3_, whose theoretical capacity can be as high as 480 mAh/g for *x* = 0. Currently, its laboratory record has reached 400 mAh/g and industrially reached 300 mAh/g—still far from the limit.

For anode materials, there is also developing space. The commonly used anodes include graphite anode and Si-based anode. The theoretical capacity of Si anode can be as high as 4200 mAh/g. However, it faces the great challenge of volumetric expansion. Once this challenge has been overcome, the anode performance will take another step forward.

Moreover, if we can develop a Li-containing anode, the cathode can be conversely Li-free, so that many more Li-free materials would become cathode candidates, thus opening new chances for LIBs.

For the other performance indices of LIBs, such as the cyclability, charge–discharge rate, temperature adaptability as well as thermal stability, we either do not yet know where the limits are or they are still very far from the limits. So, from these facets, it would be more difficult to say that we are approaching the limits.

LIBs still have many possibilities. We can continuously improve their energy density and other performances by discovering and developing different materials. There are many challenges to overcome and much research work to do before we can reach the limits.

**Chen:** LIB is a relatively complex system mainly composed of cathode, anode, electrolyte and separator. The capacities of some of the commercialized electrode active materials, such as LiCoO_2_ cathode and graphite anode, are reaching their limits. But there will be further improvement with the continuous development of new materials.

The major direction of LIBs is the improvement of the energy density of electrode materials and the overall performance of batteries. A high-capacity cathode, which is currently the determinate of battery performance, is the key and it is also important to develop anodes, electrolytes and battery-manufacturing techniques matching the cathodes. I think the short-term research and development goals of LIBs should be: energy density reaching 300–350 Wh/kg, relatively high charge–discharge rate, usable in the temperature range of –30–60°C, cycle life at normal temperature over 1500 times and pack cost <0.6 RMB/Wh.

There will be further improvement with the continuous development of new materials.

—Jun Chen

**Sun:** We have been developing LiCoO_2_ and Li(NiCoMn)O_2_ for many years, so these two systems are now relatively mature. But, through the developing history, we should notice that the pace of improvement is getting slower and slower, which means that the problems we are facing are becoming harder and harder to solve.

To overcome the current difficulties, maybe we should come back to the basic scientific questions and mechanisms of LIBs. If we can better describe the mechanism of batteries by mathematical and physical models, it would help us to further approaching the limits of LIBs.

Moreover, industrially speaking, a battery is a systematical product. Better basic theories can help us to better predict the impact of increased energy density on other indices of LIBs, including but not limited to the costs and safety.

**Cheng:** I agree that there are still spaces for LIBs to develop. And I think the future improvements may come in three directions: first, to improve the existing materials; second, to discover new materials; and, third, to develop new systems by transferring the current liquid system to semi-solid and solid systems, or other innovative systems.

## LIBS: CHALLENGES AND DIRECTIONS

**Cheng:** Currently, we cannot reproduce all the lab results in factories. So the development space of the LIB industry is even bigger than that of the LIB research.

**Zhang:** That's right. The current LIB industry systems are facing a number of challenges.

The first one is what Prof. Li has mentioned: the expansion of Si-based anodes. The anodes expand during cycling, thus the constraint force of a battery module continuously increases. Once the force exceeds the designed strength, the consequence would be catastrophic and that is what the original equipment manufacturers (OEMs) and battery manufacturers would never want to see.

The second one is the safety of the high-Ni Li(NiCoMn)O_2_ system. High-Ni materials can provide high energy density, but it is not as stable as LiFePO_4_ and low-Ni Li(NiCoMn)O_2_ materials, so its safety problem is a great challenge to be solved.

The third one is about the further improvement of LiFePO_4_. LiFePO_4_ is Co-free and has the advantages of low cost, high safety and long life. So, in recent years, it has attracted much attention from the whole industry. Our company, Gotion High-Tech, started to work on LiFePO_4_ since its foundation in 2006 and has achieved the energy density of 200 Wh/kg on the single-cell level. Right now, we are still trying to improve its performance.

The fourth challenge is the development of electrolytes with a wide temperature range of usage. Many of our clients require batteries that can be used in a broad geographic region, which means that the batteries should show high performance in a temperature range extending from –40°C to 80°C, instead of only viable at high or low temperatures. There is much work to do on electrolyte additives and solvent systems in order to produce the wide-temperature-range LIBs.

Finally, the auxiliary materials of batteries also need to improve. Besides the four major materials—the cathode, the anode, the electrolyte and the separator—the auxiliary materials including current collectors, conductive additives and binders are also important for the performance of LIBs.

**Li:** These are all key challenges for LIBs. First is the expansion of Si-based anodes. The intrinsic expansion ratio of silicon atoms after combining lithium ions is 320%. So the volume expansion has to be regulated by controlling the grain size, content of Si, microstructure, binder, electrolyte and formation process. Both particle and electrode should be designed properly.

For the safety of the Li(NiCoMn)O_2_ system, the result of the thermal runaway is mainly caused by the electrochemical reactions between the liquid electrolyte and the electrodes. So, one

We should be aware of the actual demands of the industry in our research.

—Shigang Sun

of the most promising solutions is to upgrade liquid electrolytes to solid electrolytes. However, before this switch, we can use additives, selected solvents and salts or surface coatings to stabilize the surface of the electrodes to prevent continuous side reactions with liquid electrolytes.

It is also important to strengthen the systematical basic research of Li(NiCoMn)O_2_. We should interconnect factors of heat, electricity and volume change, and give better descriptions of the levels of molecule, particle, electrode and battery cell.

We also talked about the LiFePO_4_ battery, which has been quickly developed in recent years and is now comparable to Li(NiCoMn)O_2_ in some aspects. I think its further improvement relies on adjustments to the material, such as evolving to Li(FeMn)PO_4_. Prelithiation and new anodes are also effective in improving its performances.

The auxiliary materials of LIBs are also important. Among the auxiliary materials, binder used in electrodes is critical in that it can greatly influence the cyclability of batteries. Currently, theoretical and experimental research on binder is relatively lacking. Moreover, it is not easy to quantitatively characterize the binder in a real battery because the binder amount is very small and highly dispersed at the molecular level. It is also not easy to characterize its interaction with active materials, conductive additives, current collectors and separators.

**Huang:** We need to comprehensively consider and coordinately promote the whole performance of batteries and make a balance for real applications. Among them, safety is very important but has not yet been fully understood in the sense of basic research. The battery-management system and thermal management also need to be paid more attention.

For the thermal management of LIBs, an important point is that not only the materials are critical. The thermal-control system and the circulation system are also key factors to help to modulate the temperature environment for the materials and thus extend the overall temperature range of the battery.

**Sun:** It is important to consider the application scenarios when performing battery research. To extend the temperature limit by using supplementary systems, as Prof. Huang has described, is a good example. Only by considering multiple factors not limited to the battery itself are we able to make batteries available for extreme environments such as the deep sea and deep space.

The considerations of scientists and engineers are often different. The main target of scientists is to increase the energy density, but engineers need to consider more about the comprehensive performance. So we should be aware of the actual demands of the industry in our research.

**Chen:** Battery research and industry differ greatly in their research methods and concerns. Battery research in China is mainly funded by the government, but not companies. Many Chinese companies have founded their own research institutes but they are far from perfect. So I think one of next key steps should be the tight combination of the strengths of universities and companies.

**Cheng:** The thinking modes of research and industry are actually different. Maybe Dr. Zhang can briefly describe what the industrial demands are?

**Zhang:** We have three major demands for a newly designed battery: high performance, ease of manufacture and PLM (product lifecycle management)-oriented design.

When we talk about high performance, we mean high electrochemical performance, excellent safety, good mechanical performance, as well as outstanding thermal performance. Industrially, we do not evaluate a product by a single index, but by a radar map of many indices.

We also hope for a new product to be easy to manufacture. First, no matter how excellent a material is, it cannot be made into a battery unless it is agreeable to manufacturing techniques. Second, the cost should be acceptable. Except for aerospace and other special application scenarios, all batteries should be produced in a high-quality and inexpensive way. Third, we hope the new design can be compatible with the existing production equipment, thus avoiding a waste of the past investment. Finally, the production efficiency should be high enough for mass production in a reasonable time span.

Moreover, the new design should be PLM-oriented. We should consider echelon utilization and recycling from the very beginning of the design.

Industrially, we do not evaluate a product by a single index, but by a radar map of many indices.

—Hongli Zhang

## NEW TYPES OF BATTERIES

**Cheng:** What are the promising new types of batteries?

**Chen:** Organic cathode materials are a possible direction for LIBs in the long run. These materials have high capacities and are low-cost and environmental friendly. Moreover, organic cathode materials are a rich group feasible of systematic molecular design and allow the flexible coupling of lithium-containing and lithium-free compounds. It is a promising direction still in the state of lab research, with problems of low conductivity, low energy density and solubility in organic electrolytes to be solved.

New types of batteries such as sodium-ion batteries, aqueous batteries, lithium-sulfur batteries and metal-air batteries are also promising. Among them, sodium-ion batteries and aqueous batteries are attractive for large-scale energy storage. Solidification is a major direction for electric vehicle (EV) batteries. Fuel cells are also a direction with both challenges and opportunities that has been explored for years.

**Li:** I am not sure whether introducing lithium partially as an anode can be still regarded as a kind of LIB. But no matter how LIBs are defined, replacing liquid electrolytes with solid ones is a promising direction.

The sodium-ion battery is also promising. The cost of its raw materials is rather low and its performance is nearly acceptable in many aspects. It is possible to substitute a portion of lead-acid batteries and LIBs with low energy densities in scenarios such as household energy storage, large-scale energy storage, telecommunication base stations and low-speed EVs.

There are also aluminum-ion batteries and magnesium-ion batteries. But their cyclability is poor so that it is difficult to make rechargeable batteries out of them. Personally, I am not optimistic about them for EV applications.

The lithium-sulfur battery is being widely developed. If its cyclability keeps improving, it may be possible for it to be used in scenarios in which the gravimetric energy density is more important than the volumetric energy density, such as in large-wingspan unmanned aerial vehicles and digitalized infantry equipment.

Another type of battery is the lithium-air battery, which is difficult to optimize, because it combines the difficulties of LIBs, lithium metal batteries, as well as fuel cells. The related basic research needs quite a long time to develop.

It is true that the cost of the raw materials of sodium-ion batteries is low, but it does not mean that the cost of the battery products will be low.

—Yunhui Huang

**Huang:** The sodium-ion battery is promising. But, before it can be industrialized, there are still many problems to be solved. First, we have not clarified which materials could be practically used industrially as cathode and anode materials. Second, it is true that the cost of the raw materials of sodium-ion batteries is low, but it does not mean that the cost of the battery products will be low. Currently, the cost of LIBs has been reduced to a fairly low level, so that it is a great challenge for the sodium-ion battery to reduce the cost to the extent that could be able to substitute a portion of LIBs.

It is similar for the lithium-sulfur battery, which is very promising, but its intrinsic deficiencies are still left to overcome. We have to think about how to reduce the electrolyte amount to control the overall battery volume and how to improve its safety. And I agree with Prof. Li that the lithium-sulfur battery is promising in certain scenarios, so we should conduct our research by considering these scenarios.

**Sun:** These new types of batteries belong to two major classes: one is ion batteries, including sodium-, aluminum- and magnesium-ion batteries; the second is metal batteries, including lithium-sulfur and lithium-air batteries.

The second class of batteries has higher energy density, but is facing more challenging problems, such as their poor safety and cyclability. I have a feeling that our current solutions for these problems are not fundamental, because we did not fully understand the basic mechanisms.

For example, we use physical approaches such as artificial protective films to cope with the dendrite growth on the surfaces of lithium metal electrodes. But, actually, dendrite growth is an electrochemical process of dissolution and crystallization, so the fundamental solution should be to control the nucleation and growth of the crystal. But, of course, it is currently difficult to control the crystallization of lithium metal and further explorations on the dissolution and crystallization process of lithium metal anodes in different electrolytes are needed.

So what I want to emphasize is that basic research is extremely important for the development of next-generation batteries.

**Zhang:** The industry is also very concerned about the new types of batteries. Most of the new batteries are still far from industrialization. Among them, the semi-solid battery is being developed relatively fast and we are keen to see breakthroughs soon in this field, after which the solid battery may be realized.

I agree with what the chairman of my company said—that is, the technological progress of batteries relies 50% on the progress of materials science, 30% on the progress of battery-manufacturing technologies and 20% on the progress of the design of the product systems.

Breakthroughs in materials science is the most critical driving force. We put a lot of effort into this aspect and we are investing and cooperating with universities and research institutions all over the world so that, once key discoveries appear in labs, we are able to transfer them into products as soon as possible.

Regarding the manufacturing technologies, which include the manufacture of battery cells, modules and packs, current technologies are still not good enough to fulfill the requirements of OEMs. We have to further reduce the cost, improve the safety and better match the batteries with the requirements of vehicles.

For the product system design, we are trying to explore the possibility of utilizing the entire vehicle chassis as a large integrated battery.

**Cheng:** Thank you for all your constructive opinions. I agree that, first, the breakthroughs of next-generation batteries rely largely on the breakthroughs of basic research. We have identified most of the scientific problems for the new types of batteries, on which our research should focus.

Second, our research should be more application-oriented. We should select different batteries for different applications and focus on the research that can solve specific challenges. That is how we can accelerate the development of next-generation batteries.

## BREAK THE FRAMEWORK OF ELECTROCHEMISTRY

**Cheng:** To develop practical next-generation batteries, maybe we need to be more innovative, to think about the completely new forms of batteries or energy-storage systems.

**Sun:** The existing energy-storage systems are mainly based on the conversion between chemical energy and electrical energy—that is to say, to store energy on the interface by capacitors or to store and release energy through redox reactions by batteries.

But, except for chemical energy, it is also possible to convert other energies such as biological energy, mechanical energy, solar energy and thermal energy into electricity and store it in batteries. Thus, we may be able to break the framework of electrochemistry and create new energy-storage systems.

**Chen:** The aim of energy storage is to break the temporal and spatial constraints of energy carriers so as to release energy when needed, for example, in the clean and easy-to-use form of electricity. Considering the source of energy, the ultimate choice to solve the energy crisis should be solar energy. Solar cells have been widely studied and a possible direction is bio-inspired solar cells, such as solar cells mimicking photosynthesis that can produce electricity and carbon/hydrogen-containing fuels from solar energy, water and carbon dioxide without the consumption of the low-abundance, unevenly distributed elements such as lithium and cobalt. We can also rationally design some high-energy chemical reactions to control and utilize reaction-generated energy and it may bring forth a new energy-storage form.

**Li:** Now, we store energy in the forms of electricity, heat or hydrogen. But, as long as the system is a closed one, its energy density is limited and the energy will be exhausted.

There has been a new idea to learn from organisms and create new types of energy-storage systems that are open and able to perform ‘metabolism’. For example, electric eels can intake energy through diet and convert it into electricity. As long as the eel is alive, it can eat and make electricity continuously. We may learn from it and create similar devices that can intake energy from the environment and convert it into electricity continuously. It is somewhat like a combination of fuel cells and reverse fuel cells, and can keep running with the existence of chemical fuels, solar energy, biofuel or other energy sources.

Research of this kind is relatively lacking now, but some prototype work already exist, including the work to store energy with organic reactions or the work to collect and store environmental mechanical energy. Such a dynamic open energy-storage system may be able to get rid of the energy-density limits of traditional batteries. We call them live batteries.

It [a live battery] is somewhat like a combination of fuel cells and reverse fuel cells.

—Hong Li

**Sun:** That is a good idea. In order to absorb the biological and physical factors, a big change in materials is needed. We should take biological materials and many other new materials into the current systems. Moreover, a fuel cell is a good example of an open system. It is an important energy-conversion and storage device.

**Huang:** Fuel cells and LIBs are the two major choices for EVs. They both have their own advantages and shortcomings. It is possible to combine their strengths for future development.

Actually, energy storage is a rather interdisciplinary field, touching on materials science, chemistry, electrical engineering, intelligent manufacturing, information science, mechanical engineering and even biology. Recently, the Chinese government encouraged the setting of a university major of Energy Storage

Technology, intending to promote interdisciplinary communication and foster talents for future developments in this field.

## WAITING FOR THE TAGS OF ‘INNOVATED IN CHINA’

**Cheng:** How is China's level in battery research and industry?

**Zhang:** We have compared Chinese battery products with those of Japan and South Korea, and found that our single cells are comparable to, or even better in certain indices than, the foreign ones. However, at the level of large-scale manufacture, the consistency and qualified rate of our final battery products are lower than the Japanese or South Korean ones. We have to admit that our production equipment, controlling systems and management standards are not well developed.

Regarding the domestic production equipment, we expect higher controlling accuracy, better stability and overall equipment effectiveness, which can improve our manufacturing level of battery products.

However, we do have our strengths. We are rich in raw-material resources and have built a complete industrial chain for batteries. Our production scale of the four major materials as well as the auxiliary materials is big enough to meet the domestic and export demands. If we can take full advantage of these strengths, China will become a real battery powerhouse.

**Cheng:** Would you please give some details about our production level of the four major materials?

**Zhang:** First, cathode materials. For LiFePO_4_, China has its uniqueness. We have the biggest scale of production and application. But we should notice that the key patents are not ours. For Li(NiCoMn)O_2_, our products are not as good as those of foreign enterprises. Product consistency and impurity-control techniques still need to improve.

Second, anode materials. Our production and application scales are world-leading. Chinese enterprises occupy a large share of the international market. However, in the subfield of the silicon-carbon anode, some foreign companies are doing better.

Third, electrolytes. China's production scale is huge, but the key patents of electrolyte formulas and electrolyte additives are mainly owned by foreigners. This is an urgent challenge that may cause potential risks.

It is similar for separators. Our production scale is definitely the number one in the world. China contributes >50% of the world separator market. But we do not have the key original patents.

Much work was not aimed at meeting application demands or solving key scientific problems.

—Huiming Cheng

So, generally speaking, we have a very complete battery industrial chain and a large production scale, as well as a number of world-leading material suppliers, but are still weak in intellectual properties.

**Li:** Patent is actually a big problem for China. For example, >60% of sulfide-based solid-state battery patents are owned by Japanese enterprises.

However, I think the development of next-generation batteries is a chance for China to catch up. In the past decade, many Chinese companies have been actively involved in the development of new materials and new technologies, with the number of Chinese patents growing. Universities and institutions are also very active in applying for patents, some of which are valuable innovations.

**Sun:** Chinese scientists have done a lot of work in battery research and some of them are quite influential. However, besides a few innovative achievements such as the multielectron theory proposed by Prof. Feng Wu's group, fundamental work that can be labeled as ‘Innovated in China’ is lacking.

China's publication-oriented evaluation system has brought some negative effects. We should try to come back to the scientific problems themselves, allowing the capable and thoughtful research teams to focus on basic problems for years without publication. That is the only way to foster innovative discoveries in China.

**Cheng:** That's right. We have published a lot of papers, but very few of them are rationally designed. Much work was not aimed at meeting application demands or solving key scientific problems. Either synthesizing a so-called ‘new’ material or mixing two existing materials together with electrochemical characterizations may be put into a paper for publication. This is a serious problem in China.

Moreover, most of us focus more on electrode materials, but do not pay much attention to auxiliary materials or the design of the whole-battery systems. That is what should be improved in the future.

**Li:** About 60% of the publications on batteries are written by Chinese researchers. But the advanced research methods, such as high-throughput calculation, *in situ* and Operando characterization, and digital simulation are created originally and dominated by foreign scientists.

For example, are we able to rationally design electrode materials? Scientists have proposed the Materials Genome Initiative, which aims to rationally design materials by combining high-throughput calculation, high-throughput synthesis, high-throughput characterization and big-data analysis. But, actually, there are few groups in China able to perform such kinds of activities. In order to realize the rational design of materials, how to combine the strengths of diverse disciplines and better understand the basic processes is a big question left to be discussed.

**Chen:** We should increase investment and improve the cooperation between scientists and industry in all the subfields of batteries. There should be a group of scientists focusing on basic research and original innovation, as well as a group of engineers focusing on the very details of manufacturing technologies. Only by continuous accumulation without eagerness for quick success will we be able to gain acquirements labeled as ‘Innovated in China’.

**Cheng:** Thanks for all your valuable discussions! I am personally optimistic about the future of batteries. There will be further breakthroughs and various new types of batteries will continuously support the energy basis of our society.

## Notes

Weijie Zhao is an *NSR* news editor based in Beijing.

